# Primary resistance to first- and second-generation ALK inhibitors in a non-small cell lung cancer patient with coexisting ALK rearrangement and an ALK F1174L-cis-S1189C *de novo* mutation: A case report

**DOI:** 10.3389/fphar.2022.1060460

**Published:** 2022-11-23

**Authors:** Jiuzhou Zhao, Xiang Li, Ruizhe Fan, Yaping Qin, Zhizhong Wang, Bo Wang, Shaomei Li, Jianfeng Fan, Xinxin Wu, Hongxia Liu, Yuping Guan, Yinfeng Liang, Xiao Zhang, Yongjun Guo

**Affiliations:** ^1^ Department of Molecular Pathology, The Affiliated Cancer Hospital of Zhengzhou University & Henan Cancer Hospital, Zhengzhou, China; ^2^ Henan Key Laboratory of Molecular Pathology, Zhengzhou, China; ^3^ School of Basic Medical Sciences, Academy of Medical Sciences, Henan Institute of Medical and Pharmaceutical Sciences, Zhengzhou University, Zhengzhou, Henan, China; ^4^ Department of Medical Oncology, The Affiliated Cancer Hospital of Zhengzhou University & Henan Cancer Hospital, Zhengzhou, China; ^5^ Department of Medical Imaging, Zhenping People’s Hospital, Nanyang, China

**Keywords:** non-small cell lung cancer (NSCLC), next generation sequencing (NGS), EML4-ALK, F1174L, S1189C, *de novo* mutation

## Abstract

The effectiveness of the tyrosine kinase inhibitor ALK (TKI) for non-small cell lung cancer has been confirmed. However, resistance to ALK-TKIs seems inevitable. Mutations in the ALK kinase domain have been reported as an important mechanism of acquired resistance to ALK therapy. However, patients with *de novo* ALK kinase domain mutations and ALK rearrangements who were not treated with ALK inhibitors have rarely been reported. Here, we report a case of primary drug resistance to first- and second-generation ALK inhibitors in a NSCLC patient with ALK-rearrangement. The next-generation sequencing test of the pathological biopsy showed that the *de novo* ALK kinase domain mutation F1174L-cis-S1189C may be the cause of primary drug resistance.

## Introduction

Anaplastic lymphoma kinase (ALK) gene rearrangements occur in approximately 3%–7% of patients with non-small cell lung cancer (NSCLC) (Soda et al., 2007; Takeuchi et al., 2008; Shaw and Engelman, 2013). The most common ALK fusion partner is the echinoderm microtubule-associated protein-like 4 (EML4) gene. (Katayama et al., 2015). The fusion of ALK with EML4 causes the activation of the ALK kinase domain and causes carcinogenesis (Soda et al., 2007). The discovery of the EML4-ALK fusion brought a new revolution in targeted therapy. Crizotinib is the first targeted drug approved by the FDA for ALK-positive metastatic NSCLC patients, ushering in a new era of targeted therapy for ALK-positive patients. The PROFILE 1014 (Solomon et al., 2014) study showed that crizotinib improved progression-free survival (PFS) and response rates compared with first-line chemotherapy (74% vs. 45%; *p* < 0.001). Based on FDA approval and clinical trial data, the second-generation oral ALK-TKIs alectinib, brigatinib, and ceritinib and the third-generation oral ALK-TKI lorlatinib are recommended by the NCCN guidelines for the first-line and subsequent treatment of NSCLC patients with ALK rearrangements (Shaw et al., 2016b; Ou et al., 2016; Kim et al., 2017; Peters et al., 2017; Shaw et al., 2017; Soria et al., 2017; Camidge et al., 2018; Solomon et al., 2018; Shaw et al., 2020).

Although crizotinib increases overall survival time in advanced NSCLC patients, the patients inevitably develop resistance to crizotinib. Approximately 30% of patients harboring crizotinib resistance have developed mutations in the ALK tyrosine kinase domain (Doebele et al., 2012), including L1196M (Choi et al., 2010), G1202R (Katayama et al., 2012), G1269A (Friboulet et al., 2014) and F1174L (Heuckmann et al., 2011). Although second- and third-generation ALK inhibitors have high activity in crizotinib-resistant patients, indications for the application of second- and third-generation ALK inhibitors are limited. A study pointed out that the F1174L mutation is one of the most common resistance mutations for ceritinib (Friboulet et al., 2014) and is present in approximately 16.7% of ceritinib-resistant patients (Gainor et al., 2016). The S1189C mutation in the ALK kinase domain has not been reported in previous studies, nor is it recorded in any publicly available databases of oncogenes (such as COSMIC and ClinVar). Although brigatinib (Tu et al., 2019), alectinib (Zhang et al., 2016), and lorlatinib (Syed, 2019), as FDA-approved ALK inhibitors, are effective against the F1174L mutation, previous reports have shown that the ALK gene compound mutation is resistant to almost all ALK-TKI inhibitors (Gainor et al., 2016; Takahashi et al., 2020; Salifu et al., 2022).

ALK compound mutations are mainly seen in the sequential treatment of multiple ALK inhibitors in non-small cell lung cancer patients (Yoda et al., 2018), but the case of *de novo* F1174L-cis-S1189C mutation as a primary drug-resistant mutation that appeared before targeted therapy has not been reported yet.

## Case report

**FIGURE 1:**
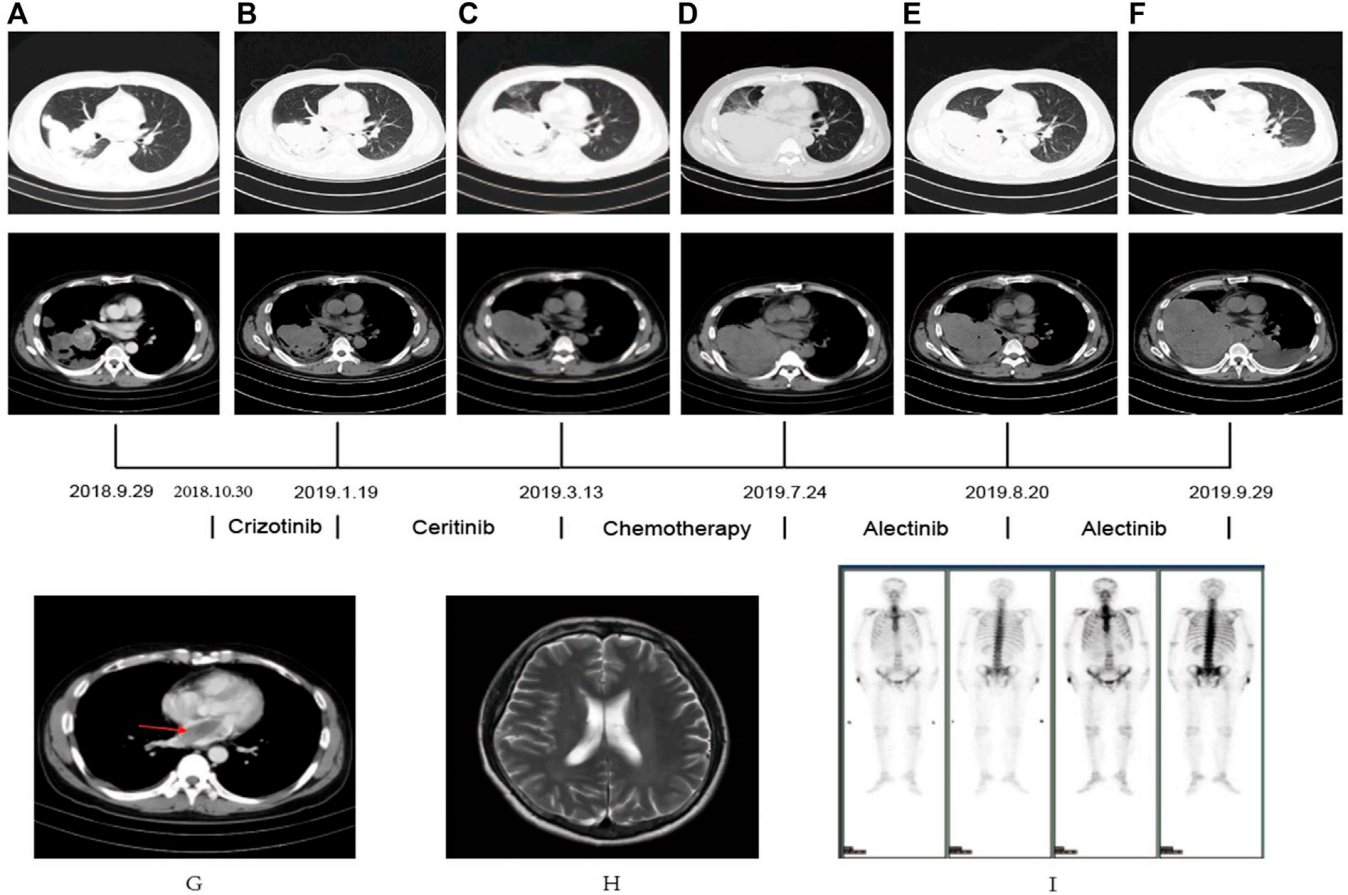
**(A–F)** CT scan showed the changes in the lung mass in the patient PRE- and POST-treatment **(G)** The CT scan revealed an atrial filling defect. **(H,I)** MRI and PET-CT showed no metastasis in brain and bone.

**FIGURE 2:**
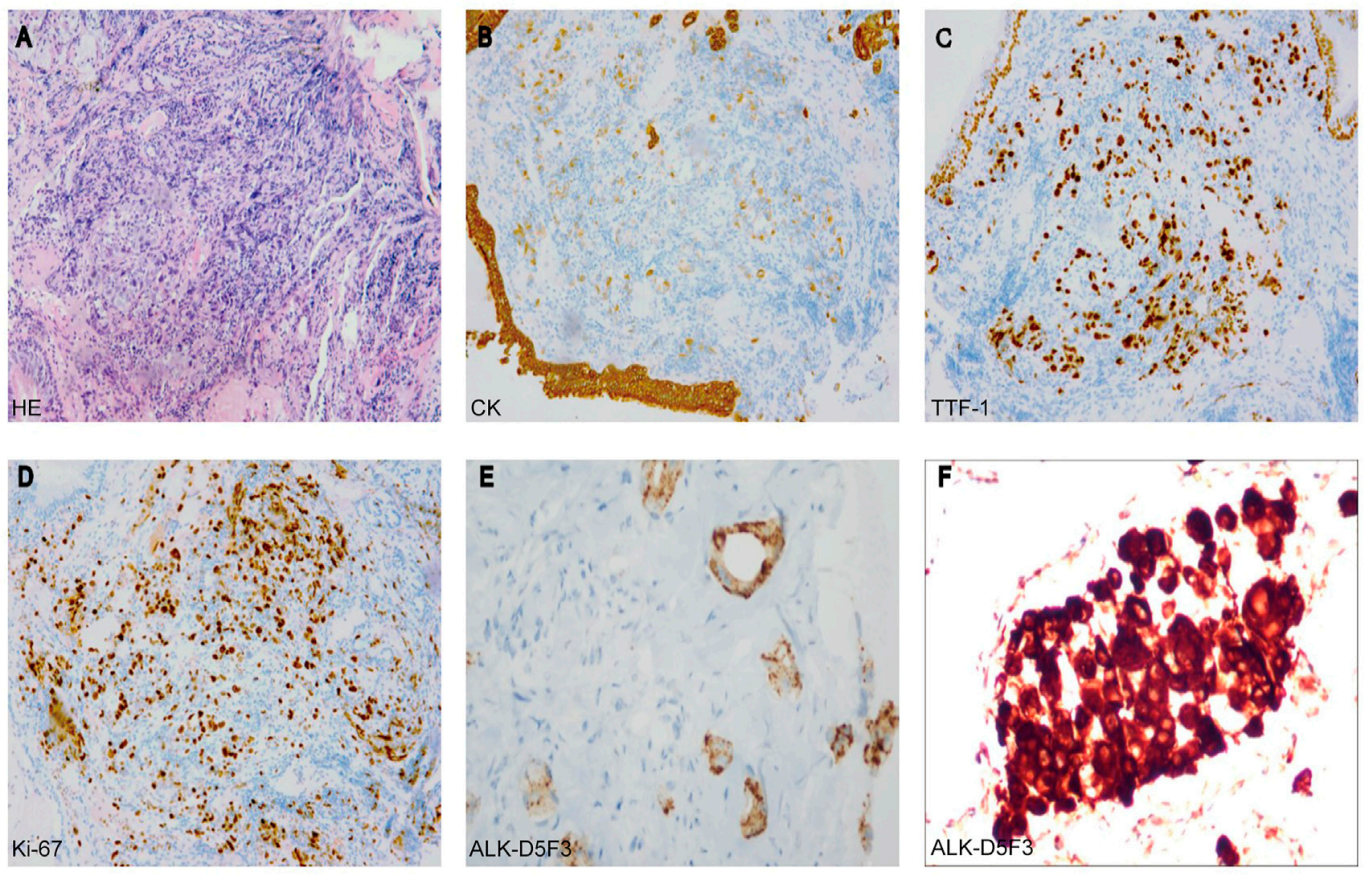
**(A)** Hematoxylin-eosin staining (HE) **(B–E)** Immunohistochemistry analysis revealed immunoreactivity to CK **(B)**, TTF-1 **(C)**, Ki-67 **(D)**, and ALK-D5F3 **(E)**. **(F)** The rebiopsy analysis revealed immunoreactivity to ALK-D5F3 **(F)**.

**FIGURE 3:**
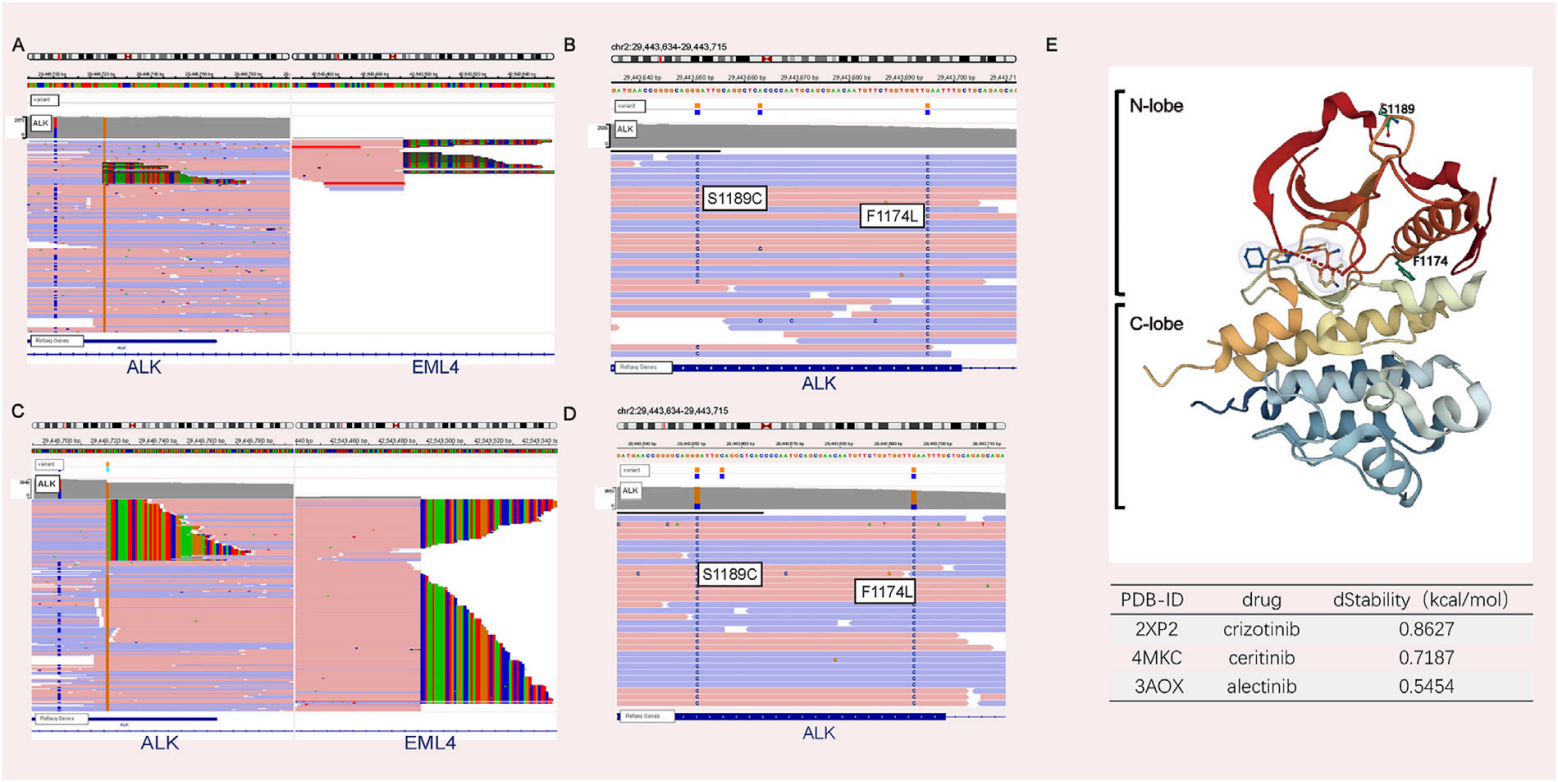
NGS analyses before and after therapy. NGS analysis before crizotinib treatment revealed the presence of the ALK fusion **(A)** plus the F1174L-cis-S1189C mutation **(B)**. NGS analysis after crizotinib and ceritinib treatment revealed the presence of the ALK fusion **(C)** plus the F1174L-cis-S1189C mutation **(D)**. **(E)** ALK structure retrieved from PDB 2XP2. *In silico* mutagenesis of human ALK binding to three ALK inhibitor complexes (PDB ID: 2XP2, PDB ID: 4MKC, and PDB ID: 3AOX) was used to predict the variable influence on protein stability. The dStability and relative thermostability of the mutation with respect to the wild-type protein are shown in the table.

A 44-year-old male patient was admitted to Henan Cancer Hospital due to cough and blood in the sputum. Chest computed tomography (CT) revealed a soft tissue mass measuring 49 mm * 70 mm in the lower lobe and hilum of the right lung, enlargement with lymph nodes in the right hilum, and irregular thickening of the right pleura with pleural effusion (Figure 1A). CT showed a filling defect in the left atrium, which was considered to be the formation of a vascular tumor thrombus (Figure 1G). No metastases of the brain or bone were found by magnetic resonance imaging (MRI) (Figure 1H) or positron emission tomography-computed tomography (PET-CT) (Figure 1I). Pathological analysis of bronchoscopy biopsy tissue confirmed lung adenocarcinoma. Immunohistochemistry (IHC) staining for ALK (Ventana-D5F3), CK, TTF-1, and Ki-67 was positive (Figures 2A–E). The presence of EML4-ALK gene fusion and an ALK exon 23 F1174L and S1189C mutation was identified by targeted next-generation sequencing (Figures 3A,B; Supplementary Methods).

The patient received crizotinib (250 mg, BID) as first-line targeted therapy for approximately 2.5 months, beginning on 30 October 2018. Based on the Response Evaluation Criteria in Solid Tumors (RECIST) 1.1 (Eisenhauer et al., 2009), progressive disease (PD) was diagnosed according to chest CT (Figure 1B), and the patient was switched to ceritinib (150 mg, QD) for nearly 2 months. However, the chest CT assessment indicated PD after ceritinib (Figure 1C), the patient subsequently underwent chemotherapy. Afterward, due to the progression of the tumor during treatment (Figure 1D), the patient underwent a rebiopsy and NGS analysis on 24 July 2019, and the result still showed EML4-ALK fusion and the F1174L-cis-S1189C mutation (Figures 2F, 3C,D). Considering the mutation status of the ALK gene kinase domain, alectinib was recommended on 24 July 2019. One month later, chest CT showed that the tumor was smaller (Figure 1E). However, his condition deteriorated again during alectinib therapy in the second month (Figure 1F), and the patient ultimately passed away in October 2019.

*In silico* mutagenesis of human ALK binding to three ALK inhibitor complexes (PDB ID: 2XP2, PDB ID: 4MKC, and PDB ID: 3AOX) was used to predict the variable influence on binding affinity and protein stability (Supplementary Methods; Supplementary Table S1). Figure 3E illustrates that F1174 is at the C-terminal end of the αC-helix and S1189 is situated at the β-turn of the N-lobe. Since compound mutations are not in contact with the ATP/drug-binding cleft, compared with the wild type, there is no obvious difference in the binding affinity between ALK and ALK-TKI. However, the F1174L-cis-S1189C mutation decreases the binding stability (dStability>0) between ALK and ALK-TKIs. Therefore, the F1174L-cis-S1189C mutation is predicted to affect drug efficacy (Jiang et al., 2018).

## Discussion

The F1174L mutation, as an acquired mutation of the ALK tyrosine kinase domain, is an important factor affecting targeted therapy. The ALK F1174L mutation was discovered in neuroblastoma (Azarova et al., 2011). In non-small cell lung cancer, the F1174L mutation has been reported as a coresistance mutation site of crizotinib and ceritinib. Studies (Sasaki et al., 2010; Heuckmann et al., 2011; Amin et al., 2016) have shown that the ALK F1174L mutation increases the affinity between ALK and ATP, leading to drug resistance in patients. At present, brigatinib, alectinib, and lorlatinib, which can overcome F1174L resistance mutations, have been approved by the FDA. The S1189C mutation, located in the ALK kinase domain (from 1,116 to 1,392, https://www.uniprot.org), has not been reported in any research or recorded in any databases. The functional effects of the ALK kinase domain are inconclusive.

Compound resistance mutations in the ALK gene are more common after resistance to second- and third-generation inhibitors, potentially due to the accumulation effect of mutations. As we showed *in silico* results, compared with the wild-type, the binding with ALK with compound mutations became more unstable. Combined with previous reports, these results suggest that compound mutations of the ALK gene affect the efficacy of first- and second-generation ALK-TKIs. To date, compound mutations of the ALK tyrosine kinase domain have been commonly considered a molecular mechanism of resistance to all-generation ALK-TKIs(Gainor et al., 2016; Yoda et al., 2018; Okada et al., 2019; Takahashi et al., 2020; Salifu et al., 2022). In only one report, the ALK C1156Y/L1198F composite mutant was found to be resistant to second- and third-generation ALK-TKIs but resensitive to crizotinib (Shaw et al., 2016a).

In this case, the diagnosis of lung adenocarcinoma was clear. In addition, the patient had undergone genetic testing that confirmed indications for targeted therapy, and crizotinib has become the first choice for targeted therapy. However, this patient had a poor effect during the application of crizotinib and ceritinib. The reasons for this effect need to be clarified. It is worth noting that the patient’s first diagnosis test report showed that in addition to the EML4-ALK fusion mutation, the patient also had the ALK F1174L and S1189C mutations. This may be the reason why this patient progressed within a short period after using crizotinib and ceritinib. Previous studies (Song et al., 2015) have shown that alectinib is effective for patients with F1174L resistance mutations. Since the S1189C mutation of the ALK gene has not been documented in previous studies and databases, treatment with alectinib was attempted for this patient. During the first month of alectinib treatment, the patient’s symptoms resolved but soon progressed again.

In this case, clinical targeted therapy had a poor curative effect on the patient, and this experience in clinical treatment is worth analyzing and summarizing. The patient’s gene mutation status before clinical treatment showed that the *de novo* ALK F1174L-cis-S1189C mutation and the EML4-ALK fusion mutation existed before targeted therapy, and the primary drug resistance mutations of the gene affected clinical treatment strategies and the patient’s prognosis. Genetic testing for the detection of primary drug resistance mutations is important. The role of NGS technology in preclinical research and clinical applications is highly valuable in genetic testing. Compared with IHC (ALK-D5F3, Ventana) and FISH, NGS can detect both rearrangements and mutations of the ALK gene. Therefore, NGS can accurately detect gene mutation sites and facilitate the screening of mutations that may affect the clinical treatment and prognosis of patients.

ALK *de novo* mutations are uncommon in kinase inhibitor-naïve ALK-rearranged lung cancers. A literature review revealed that only one previous study reported a patient with ALK rearrangement with a primary S1206F mutation (Lucena-Araujo et al., 2016). Patients with primary compound mutations have not been reported. In addition, no clinical benefit was observed in this patient. This may be related to the coexistence of the F1174L and S1189C mutations. Therefore, NGS detection can provide more comprehensive molecular information for clinical practice.

## Conclusion

In summary, we found a patient with ALK rearrangement plus a *de novo* ALK F1174L-cis-S1189C mutation in kinase inhibitor-naïve lung cancers. NGS detection can effectively help in the clinical diagnosis of ALK fusion plus kinase domain mutations. In this case, clinical efficacy observation showed that either the first-generation inhibitor crizotinib or the two second-generation inhibitors with different molecular parent nuclear structures, ceritinib and alectinib, showed poor therapeutic effects. This is first report of the *de novo* ALK F1174L-cis-S1189C mutation. The molecular mechanism of resistance to ALK inhibitors is still unclear. Therefore, further studies to investigate the effect of such compound mutations on different ALK inhibitors are needed to more effectively treat patients with ALK F1174L-cis-S1189C mutations.

## Data Availability

The datasets for this article are not publicly available due to concerns regarding participant/patient anonymity. Requests to access the datasets should be directed to the corresponding author.
