# Supplementary material for: Primary resistance to first- and second-generation ALK inhibitors in a non-small cell lung cancer patient with coexisting ALK rearrangement and an ALK F1174L-cis-S1189C de novo mutation: A case report
Source: Front Pharmacol. 2022 Nov 23;13:1060460. doi: 10.3389/fphar.2022.1060460 (PMC9727108; doi:10.3389/fphar.2022.1060460)
Supplement: Supplementary file 1 [file DataSheet1.ZIP › Supplementary/Supplementary methods.docx]

**NGS Library Preparation and Sequencing**

The tissue DNA was extracted using QIAamp DNA formalin-fixed paraffin-embedded (FFPE) tissue kit (Qiagen, USA). A minimum of 50 ng of DNA is required for NGS library construction. The tissue DNA was sheared using Bioruptor plus (Diagenode, Belgium), then the sheared DNA underwent end repaired and adaptor ligation. The sheared and purified genomic DNA products were amplified to form a pre-capture library. The final library was subsequently obtained by capture and purification using hybridization reactions. Briefly, genetic profiles of tissue samples were assessed by capture-based targeted deep sequencing using the 8-gene panel (Burning Rock Biotech Ltd. Guangzhou, China), covering 76kb of human genome, including eight genes: EGFR, ALK, BRAF, ERBB2, KRAS, MET, ROS1, and RET. All indexed samples were sequenced on a NextSeq 550 (Illumina, Inc., USA) with pair-end reads.

**Molecular Modeling**

In silico mutagenesis of human ALK Binding to three ALK inhibitors complex (PDB ID: 2XP2, PDB ID: 4MKC, PDB ID: 3AOX) were used to predict the variable influence on binding affinity and protein stability. The proposed residue sites were substituted to 19 other amino acids and an ensemble of the conformations (the number of conformations was limited to 25), was generated for each mutant by low-mode MD (Molecular Dynamics), the parameters we used including iteration limit 50, RMSD limit 0.25, energy window 10, conformation limit 25, fix residues farther than 4.5, 0 tether sidechains and one tether backbone. MM/GBVI was applied to calculate the binding affinity of each conformation and ALK molecules. The force field used for calculation was OPLS-AA, and the implicit solvent was the reaction field (R-Field) model. All calculations were performed in MOE (2020) (Molecular Operating Environment) software.
